# The Production of Water Kefir Drink with the Addition of Dried Figs in the Horizontal Rotating Tubular Bioreactor

**DOI:** 10.3390/foods13172834

**Published:** 2024-09-06

**Authors:** Mladen Pavlečić, Mario Novak, Antonija Trontel, Nenad Marđetko, Vlatka Petravić Tominac, Ana Dobrinčić, Monika Kralj, Božidar Šantek

**Affiliations:** Department of Biochemical Engineering, Faculty of Food Technology and Biotechnology, University of Zagreb, Pierottijeva 6, 10000 Zagreb, Croatia; mnovak@pbf.hr (M.N.); atrontel@pbf.hr (A.T.); nmardetko@pbf.hr (N.M.); vpetrav@pbf.hr (V.P.T.); adobrincic@pbf.hr (A.D.); mokralj1307@gmail.com (M.K.); bsantek@pbf.hr (B.Š.)

**Keywords:** water kefir production, functional drink, horizontal rotating tubular bioreactor (HRTB), dried fruits addition

## Abstract

Water kefir is a product obtained through the fermentation of sucrose solution, usually with some kind of dried fruit addition, by a combined culture of micro-organisms which are contained within kefir grains. Its popularity is rising because of the simplicity of its preparation and its anti-inflammatory, antioxidant, probiotic, and antibacterial effects. In this research, the water kefir production was studied in 250 mL jars, as well as in a horizontal rotating tubular bioreactor (HRTB). The first part of the research was conducted in smaller-scale (jars), wherein the optimal fruit and fruit portions were determined. These experiments included the addition of dried plums, apricots, raisins, dates, cranberries, papaya, and figs into 150 mL of initial sugar solution. Also, the optimal ratio between dried fruit and sucrose solution (0.2) at the beginning of the bioprocess was determined. The second part of this research was conducted using HRTB. The experiments in the HRTB were carried out by using different operational modes (constant or interval bioreactor rotation). A total of six different bioreactor setups were used, and in all experiments, figs were added at the beginning of the bioprocess (0.2 ratio between dried figs and sucrose solution). On the basis of the obtained results, the interval bioreactor rotation mode proved to be the better HRTB mode for the production of the water kefir, as the yield of the main fermentation products was higher, and their ratios were the most adequate for the quality of water kefir drink. The optimal results were obtained via HRTB setup 3/57 (3 min rotation, 57 min pause within 1 h) and rotation speed of 3 rpm. Furthermore, it is clear that HRTB has great potential for water kefir production due to the fact that HRTB experiments showed shorter fermentation times (at least five times) than water kefir production in jars.

## 1. Introduction

Water kefir is a fermented beverage obtained via the fermentation of an aqueous solution of sucrose by micro-organisms present in water kefir grains. Micro-organisms that are responsible for product formation are mostly immobilized in water kefir grains, and these communities comprise 6–16 different types of micro-organisms [1]. The most abundant species are lactic acid bacteria (LAB), yeasts, predominately *S. cerevisiae*, acetic acid bacteria, and *Bifidobacteria* [2,3]. The whole process of the isolation and identification of micro-organisms is complex, and for water kefir, micro-organism species, and subspecies determination, shotgun metagenomics was used [4,5,6]. Water kefir is similar to the more familiar milk kefir, but there are some minor differences. In comparison to milk kefir, which provides significant amounts of protein as well as probiotics and prebiotics, water kefir can be an interesting alternative as a probiotic and prebiotic source for vegans and people who are allergic to dairy products. It is also important to say that both of these grains are important due to their unique health benefits. 

For water kefir production, usually, dried fruit is added at the beginning of the bioprocess as it can serve as an additional source of growth- and metabolism-stimulating compounds for fermenting micro-organisms [7]. So far, it has been shown that the addition of different types of fruits can be used; however, the addition of dried figs has shown the best-quality results [8]. The exact reason for this is not fully understood, but in comparison to the addition of other fruits like dried plums, raisins, or dates, the addition of figs greatly modified the fermentation rate and had a significant impact on lactic and acetic acid concentrations in water kefir [9]. Also, a greater mass net increase in water kefir grains was recorded when figs were added [8]. One of the possible explanations could be the fact that figs, among all other dried fruits, contain the highest amount of growth-promoting calcium, which can also act as a buffer in water kefir. The calcium contents in some fruits are as follows: dried (162 mg of Ca/100 g of fruit) and raw (35 mg of Ca/100 g of fruit) figs; dried apricots (55 mg of Ca/100 g of fruit); dried papaya (54 mg of Ca/100 g of fruit); raisins (50 mg of Ca/100 g of fruit); dried dates (39 mg of Ca/100 g of fruit); and raw plums (19 mg of Ca/100 g of fruit) (data source: USDA FoodData Central (FDC); fdc.nal.usda.gov). The calcium ions actually prevent water kefir from becoming too acidic and, thus, reduce acidic stress for the working micro-organisms [10]. Calcium ions can also neutralize the negative effect of low pH levels on kefir grain growth via their indirect impact on the activity of lactic acid bacteria glucansucrases, which are directly associated with water kefir grains’ growth [10]. The fermentation itself can take place at temperatures between 20 and 37 °C, but the optimal temperature is between 20 and 25 °C. It usually lasts 2–4 days, and after the bioprocess has finished, kefir grains can be separated from the supernatant, rinsed, and then used for a new fermentation process. The end result of the fermentation process is a slightly sweet and sour beverage that may contain small amounts of ethanol and acetic acid [7]. The sweetness of the final product comes from residual sugar and acidity from organic acids produced by lactic and acetic acid bacteria [11]. The interesting part is that there is no identical kefir grain, meaning that, all things being equal, the final drink composition is always different when different grains are being used. Organoleptic inconsistency is one of the reasons for small industrial production. So far, little research has been performed at a larger and more-controllable scale [12,13]. 

In this research, water kefir production was studied in two phases. In the first phase of research, the impact of the addition of different dried fruits on water kefir production (raisins, cranberries, papaya, apricots, dates and figs) was studied in jar containers. Based on the obtained results, it was clear that figs are the most suitable dried fruit for water kefir production. In our further experiments, optimal fig quantity was determined in order to obtain desirable water kefir quality (ratios of final ethanol and lactic and acetic acid concentrations). It was observed that the initial ratio between dried figs and a sucrose solution of 0.2 gives the best water kefir quality. Consequently, this initial ratio between the dried figs and the sucrose solution was used in the second part of our investigation. This research phase was characterized by HRTB experiments. As far as we know, HRTB has never been used for water kefir production, which is the novelty of this research. During the second phase of our research, the impact of HRTB operational conditions—i.e., bioreactor rotation speed and bioreactor rotation mode (constant or interval)—on water kefir production was studied. Based on the obtained results, it is clear that HRTB has great potential for water kefir production.

## 2. Materials and Methods

### 2.1. Water Kefir Grain Maintenance and Inoculum Preparation

In all of our experiments, water kefir grains (home crafted, Zagreb, Croatia) that have been used came from static cultivation. The medium that was used for water kefir grains maintenance and propagation was prepared by dissolving 60 g of sucrose (Croatian Sugar Industry, Županja, Croatia) in 2 L of water in a 3 L Erlenmeyer flask. At the beginning of the bioprocess, several undamaged dried whole figs (local market, Zagreb, Croatia) were added, and the flask was kept at 25 °C for 3–4 days. After this time, the medium was replaced with a fresh one, and the water kefir grains were rinsed with tap water before they were returned to fresh media. These kefir grains were used for all the experiments in this investigation.

### 2.2. Water Kefir Production in Jars with the Addition of Different Dried Fruits

During the first phase, the impact of adding various dried fruits at the beginning of water kefir production was studied. Several dried whole fruits were tested, including raisins, cranberries, figs, pitted dried apricots, papaya, dates, and plums (Bio&Bio health food store, Zagreb, Croatia). All of the aforementioned dried fruits were sourced from a local store except for the whole dried figs, which were obtained from a local producer. The experiments were conducted in 250 mL glass jars, each containing 150 mL of a sucrose solution (60 g/L). In total, 6 batches of jars were prepared, with each batch containing one type of dried fruit (10 g) along with 15 g of water kefir grains. In total, at the beginning of research, 60 jars were prepared. The jars were incubated at 25 °C and each jar was loosely sealed with a screw cap to allow the gradual release of fermentation gasses. Based on the initial results, additional experiments were conducted using the same setup (with an additional 20 jars), but with higher quantities of dried figs, specifically 20 g and 30 g. This was performed in order to optimize the amount of added dried figs for the improvement of water kefir quality and bioprocess efficiency. The content of each jar was analysed every 3–4 days, so the whole bioprocess of water kefir production was monitored over a period of 32 days in order to ensure that all substrates were completely consumed. Supernatant analysis was performed using UPLC to determine the concentrations of substrates and products. Throughout the study, pH changes were also monitored. 

### 2.3. Water Kefir Production in the Horizontal Rotating Tubular Bioreactor (HRTB)

After the screening process in glass jars, the second phase of this research was conducted using a horizontal rotating tubular bioreactor (HRTB; Rosing d.o.o., Zagreb, Croatia) by adjusting operational conditions, specifically, rotation speed and rotation mode. The HRTB is made of stainless steel, and it consists of a horizontal cylindrical vessel placed on bearings that enable the rotation of the whole bioreactor. The bioreactor is 0.60 m long, with an internal diameter of 0.25 m. Inside the vessel, there are two opposing paddles measuring 0.04 m in height, running the full length of the cylinder to ensure more efficient mixing. When the constant rotation mode was used, the bioreactor rotation speed was set to 3, 5, and 7 rpm, respectively. Interval rotation mode means that the HRTB rotated for specific periods (3, 6, or 9 min) within each hour, with the remaining time (57, 54, 51 min) spent at rest (no rotation). Based on the results obtained in the research with constant HRTB rotation mode, the experiments with interval HRTB rotation mode were conducted at a rotation speed of 3 rpm. In all experiments (both constant and interval rotation modes), an initial 1 L of sucrose solution (30 g/L) was inoculated with 100 g of water kefir grains, and 200 g of dried figs (15.4% *m*/*v*) were added resulting in a total broth volume of approximately 1.5 L in the HRTB. The initial mass of dried figs (200 g) in the HRTB was determined based on the jar experiments where a ratio of 0.2 between the mass of dried figs (30 g) and the sucrose solution (150 mL) produced the best water kefir quality (Table 1). Therefore, the same ratio of figs to sucrose solution (200 g to 1000 mL) was also used in HRTB experiments. During this research phase, only the supernatant was analysed by sampling 2–3 times per day for UPLC analysis. The samples were used to monitor the changes in substrates and products concentrations. All samples were taken through the sampling valve of the HRTB. All experiments in the HRTB were performed at room temperature (around 22 °C), and pH changes in the medium were monitored throughout the experiments. However, pH monitoring was sometimes challenging due to the formation of a mash in the bioreactor, caused by the interaction between the kefir grains and dried figs during HRTB rotation.

### 2.4. Liquid Sample Analysis

For the purposes of this research, throughout all experiments, liquid samples of the media were analysed to determine the concentrations of the substrates and the fermentation products using UPLC analysis. The first step in sample preparation involved the addition of salt a solution (100 g/L of ZnSO_4_) in a 1:1 ratio with the original samples to precipitate proteins. A 500 µL aliquot of the liquid portion of the samples was pipetted into two Eppendorf cuvettes, followed by the addition of 500 µL of zinc sulphate heptahydrate (Kemika d.o.o, Zagreb, Croatia) solution. This was followed by homogenization using a vibro-mixer for 15 s, after which the samples were left to rest for 10 min. The samples were then centrifuged for 10 min at 12,225 g in a centrifuge (CF-10 Witeg, Witeg Labortechnik GmbH, Wertheim, Germany) to separate the supernatant from the solid portion of the samples. After centrifugation, 750 µL of supernatant from each cuvette was transferred to a new cuvette, and the supernatant was further diluted in a 1:1 ratio with demineralized water. Samples prepared in this manner were filtered through a 0.20 µm nylon syringe filter (Macherey-Nagel GmbH & Co. KG, Düren, Germany) into vials. The concentrations of glucose, fructose, sucrose, lactate, acetate, ethanol, glycerol, and mannitol in samples were determined using UPLC on an Agilent Technologies 1290 Infinity II LC system (Santa Clara, CA, USA) equipped with a Carbo-H++ precolumn (4 mm × 3 mm; Phenomenex, Des Plaines, IL, USA), a Rezex ROA column (15 cm × 7.8 mm; Phenomenex, Des Plaines, IL, USA), and a refractive index detector (RID). The injection volume was 10 µL. A 0.0025 M H_2_SO_4_ (Kemika d.o.o, Zagreb, Croatia) solution was used as the mobile phase at a flow rate of 0.6 mL min^−1^. The temperature of the column was set to 30 °C. Dana analysis was performed using OpenLab CDS software (Rev. C. 01. 08 [210]). 

### 2.5. Statistical Analysis of Experimental Data

During the study of water kefir production in jars, all experiments were repeated at least twice, and the standard deviation of all measurements fell within the range of experimental error (<4.9%). In the HRTB investigation, all experiments were also repeated at least twice, with the standard deviation of all measurements within the experimental error range (around 3.8%). The standard deviation of the experimental data was calculated using the standard procedure in the software Statistica 12.0 (StatSoft; Tulsa, OK; USA).

## 3. Results

**Figure 1:**
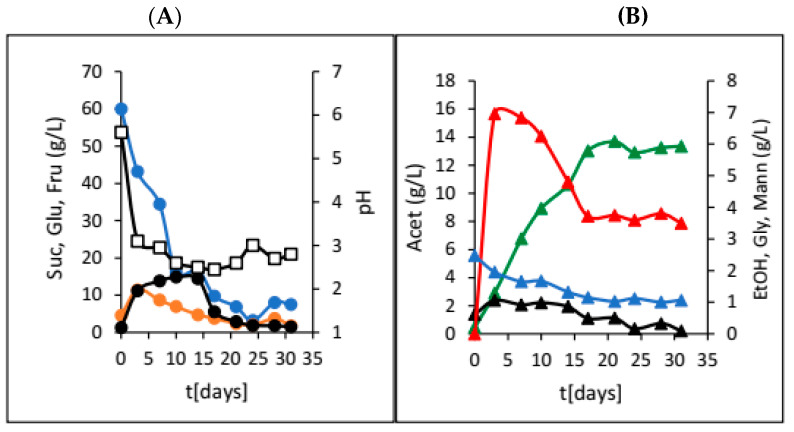
Changes in concentration of sugars and pH (**A**) sucrose (●; Suc), glucose (●; Glu), fructose (●; Fru), and pH (**□**) and fermentation products (**B**) ethanol (▲; EtOH), glycerol (▲; Gly), acetic acid (▲; Acet), and mannitol (▲; Mann) during water kefir production in jars with the addition of 10 g of dried pitted plums.

In this section, all results obtained during both jar and HRTB experiments are presented. Figure 1 shows the results from water kefir production with the addition of dried pitted plums.

**Figure 2:**
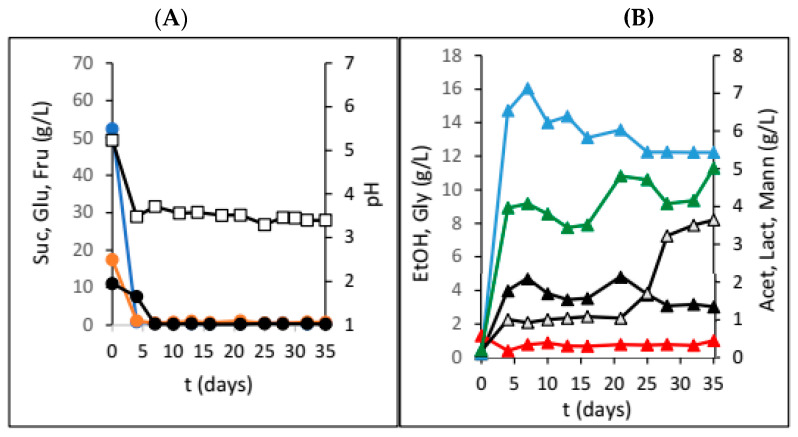
Changes in concentration of sugars and pH (**A**) sucrose (●; Suc), glucose (●; Glu), fructose (●; Fru), and pH (**□**) and fermentation products (**B**) ethanol (▲; EtOH), glycerol (▲; Gly), lactic acid (△; Lact), acetic acid (▲; Acet), and mannitol (▲; Mann) during water kefir production in jars with the addition of 30 g of dried figs.

Figure 2 illustrates the changes in the concentrations of all carbon sources, along with the concentrations of products and corresponding pH value changes, when whole dried figs were used.

Additionally, different types of dried fruits were used in this series of experiments. Results obtained in this part of the research are shown in Table 1.

**Figure 3:**
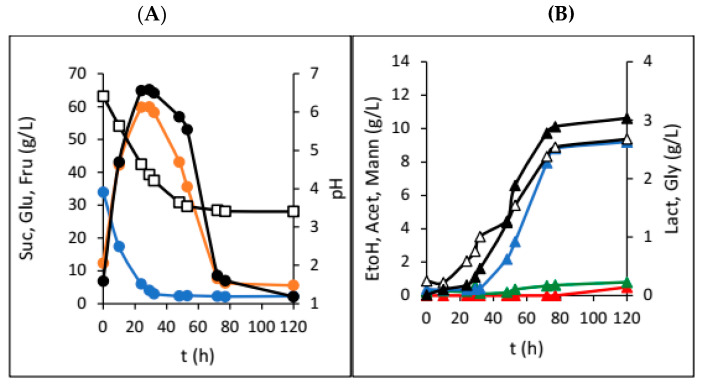
Changes in concentration of sugars and pH (**A**) sucrose (●; Suc), glucose (●; Glu), fructose (●; Fru), and pH (**□**) and fermentation products (**B**) ethanol (▲; EtOH), glycerol (▲; Gly), lactic acid (△; Lact), acetic acid (▲; Acet), and mannitol (▲; Mann) during water kefir production in the HRTB with constant HRTB rotation mode and the rotation speed of 3 rpm.

Figure 3 displays the results from water kefir production in the HRTB under constant HRTB rotation mode with the rotation speed set to 3 rpm.

**Figure 4:**
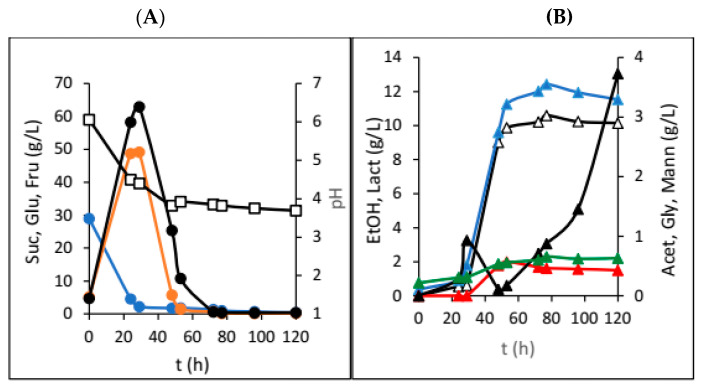
Changes in concentration of sugars and pH (**A**) sucrose (●; Suc), glucose (●; Glu), fructose (●; Fru), and pH (**□**) and fermentation products (**B**) ethanol (▲; EtOH), glycerol (▲; Gly), lactic acid (△; Lact), acetic acid (▲; Acet), and mannitol (▲; Mann) during water kefir production in the HRTB with interval HRTB rotation mode (3 min rotation, 57 min stationary) and rotation speed of 3 rpm.

The last set of experiments in the HRTB was conducted using interval rotation mode, where the HRTB rotated for a set period within each hour, with the remaining time at rest. Here, the HRTB rotation speed was set to the lowest possible value (3 rpm) because, in the experiments with constant rotation mode, we observed the deterioration of the water kefir grains and figs, along with the formation of mash. Figure 4 shows the results obtained in an experiment where the HRTB operated in interval rotation mode for 3 min with a rotation speed of 3 rpm.

The summarized results of water kefir drink production in different HRTB operational conditions are presented in Table 2. 

## 4. Discussion

The HRTB was originally designed for anaerobic bioprocesses, as well as aerobic bioprocesses with relatively low oxygen requirements (e.g., simultaneous nitrification and denitrification of wastewater). The main goal of this research was to evaluate the potential of the HRTB for water kefir production together and to determine its optimal operational mode (constant or interval rotation mode). Therefore, the primary focus of this research was to assess the performance and efficiency of water kefir production in the HRTB and to compare it with another bioreactor system. During this study, two different sets of experiments were performed. In the first phase of this research, the impact of adding various dried fruits on the dynamics and efficiency of water kefir production was investigated. For this reason, six different dried fruits were added at the beginning of the fermentation process. So far, different experimental setups have been used for the study of water kefir production, such as in examining the effects of initial nutrients and oxygen concentrations on water kefir composition [8]. Other studies have explored different initial substrates, including sucrose, molasses, honey, and various fruit and vegetable juices [2]. Recently, water kefir production using water-soluble coconut extract [14], tigernut milk [15], passion fruit [16], pineapples [17], hazelnut milk [18], and red pitaya [19] were investigated. In addition to determining which micro-organisms in water kefir contribute to the best kefir composition and quality and comparing kefir production dynamics, Kohler et al. [13] also studied water kefir production in a stirred tank bioreactor. As mentioned previously, the goal of this phase of research was to determine the optimal fruit type and dosage for water kefir production. When dried plums were used, we observed overall slower sucrose consumption (Figure 1), higher mannitol and acetic acid concentrations, and a rapid initial pH drop. This was in accordance with fermentations preformed on the media with lower concentrations of growth- and metabolism-stimulating compounds [8]. Furthermore, lower concentrations of glycerol and ethanol were also detected, which is consistent with indicating that fermentations with water kefir grains in media with low stimulating compounds result in a higher ratio of acetic acid to ethanol and acetic acid to lactic acid [8]. This outcome is attributed to the prevalence of micro-organisms such as the yeast *Dekkera bruxellensis* and the bacteria *Lactobacillus hilgardii*, which have been previously reported in such fermentations. The observed product ratios are due to the fact that heterofermentative lactic acid bacteria generally produce more mannitol compared to homofermentative strains [20], which was also observed in this research (Figure 1). Due to the relatively fast production of acetic and lactic acid in the first five days, a fast drop in the pH value (3 pH units) was observed, and this value did not change much till the end of the bioprocess (Figure 1A). After this time, an increase in acetic acid was observed, coupled with a decrease in mannitol concentration because of acetic acid bacteria activity. It is well known that acetic acid bacteria can oxidize different carbon sources, including mannitol, to obtain energy, leading to a reduction in mannitol concentration in the medium [21]. Similar results were obtained during our research with raisins, apricots, papaya, cranberries, and dates (Table 1). These fermentations also followed the concentration profiles typical of media with low levels of growth- and metabolism-stimulating compounds. In the majority of the experiments, higher concentrations of acetic acid and mannitol and lower concentrations of ethanol and glycerol were obtained. Sucrose was almost totally consumed only in the experiments with plums and the experiments with figs. However, the results of experiments with dried figs were not straightforward. In Table 1, the final concentrations of sucrose, glucose, fructose, and most of the products of microbial activity are presented. As can be seen in Table 1, the presence of lactic acid in water kefir was only observed in experiments with the addition of dried figs. This indicates that lactic acid bacteria did not have optimal conditions for growth and lactic acid synthesis in media containing other fruits. Therefore, lactic acid production was significantly reduced to concentrations below the detection limits of the chromatographic method. Furthermore, lactic acid bacteria were also affected by these conditions. Although lactic acid bacteria were impacted by these conditions, they remain an integral part of the water kefir grains due to their protective role. Water kefir grains are composed of approximately 45% mucopolysaccharide, 34% proteins, and 4% fats, along with the group B vitamins, vitamin K, calcium, phosphorous, and magnesium [22]. Figs are known as a very rich source of different growth- and metabolism-stimulating compounds, as well as their buffering capacity, and therefore, they are the preferred dried fruit for water kefir production. However, when only 10 and 20 g of dried figs were added, we observed almost identical experimental results that matched fermentations on media with low concentrations of growth- and metabolism-stimulating compounds. In these two experiments, the highest acetic acid concentrations were recorded compared to all experiments in the first phase of this research. However, the highest ethanol concentrations were obtained in the experiments where 30 g of figs was added at the beginning of water kefir production (Figure 2). As can be seen in Figure 2B, a significant increase in ethanol and acetic acid concentrations was observed in the first five days of the bioprocess, which correlated with substantial sugar consumption. This occurrence can be attributed to increased yeast activity due to the enrichment of the medium with growth- and metabolism-stimulating compounds extracted from figs. Under these conditions, yeasts have the capacity to compete with acetic acid bacteria, leading to higher ethanol concentrations. At the same time, lactic acid bacteria activity was also present, which shows that the cultivation medium contains sufficient amounts of different compounds that stimulate their growth and activity. However, lactate concentration did not increase significantly. The reduction in ethanol concentration (due to its oxidation by acetic acid bacteria into acetate) [21] and the oscillation in acetic acid concentrations allowed lactic acid bacteria to create conditions advantageous to increased lactate production. The highest lactate concentration was obtained at the end of the bioprocess, which improved the water kefir quality (Figure 2B). This increase in lactate concentration can be a consequence of sugar extraction from the added figs (30 g of dried figs contain approximately 14.4 g of fermentable sugars) (Data source: USDA FoodData Central (FDC); fdc.nal.usda.gov, https://fdc.nal.usda.gov/fdc-app.html#/food-details/746768/nutrients (accessed on 20 August 2024)). The lactate concentration increased after 20 days of cultivation (Figure 2B). Based on this fact, it is only possible to make a rough estimation of sugar extraction efficiency from the figs (in the range of 30–40% total sugar content). This estimation assumes a sugar conversion into lactate of 1 g/g by homofermentative or 0.5 g/g by heterofermentative lactic acid bacteria [23]. It is evident that lactate, with an increase of approximately 2.5 g/L, was produced from the sugars extracted from the figs. It is important to note that sugar extraction from dried fruits will be the focus of our future research. In summary, in nearly all experiments from the first phase of this research, the concentrations of acetic acid were considerably high, suggesting that fermentation conditions likely favoured the dominance of heterofermentative bacteria over homofermentative bacteria [24]. Furthermore, there is also the possibility that the acetic acid bacteria, which are naturally present in water kefir grains, contributed to total acetic acid concentration [24]. The absence of an additional nitrogen source could be the reason for acetic acid bacteria dominance during the first phase of this research. However, experiments involving different amounts of dried figs demonstrated that even minimal additional supplies of growth or metabolism-stimulating compounds allow other members of the microbial consortium, such as yeasts and lactic acid bacteria, to compete with acetic acid bacteria for substrates, thereby improving the final water kefir composition and quality. After the first phase of the research, a series of experiments in the HRTB on different substrates, initially designed for bioprocesses in anaerobic and semi-aerobic conditions, were conducted. As stated previously, the initial ratio between the dried figs and the sucrose solution at the beginning of each fermentation was 0.2. The inoculation was performed by adding 100 g of the water kefir grains. In total, six experiments were conducted by manipulating the bioreactor operational conditions. Figure 3 shows the dynamics of water kefir production in the HRTB with constant rotation at 3 rpm. During this experiment, the fastest sucrose hydrolysis was observed, and consequently, the highest initial glucose and fructose concentrations were detected compared to the other two experiments with constant HRTB rotation. The largest portion of sucrose was hydrolysed after 30 h of fermentation, and the peak concentrations of all products were recorded around 78 h of fermentation. The higher initial concentrations of glucose and fructose likely originated from the media used to maintain the kefir grains, which was partially introduced into the HRTB during inoculation, as well as from the added figs. The liberation of sugars and other compounds from the added figs is a consequence of the bioreactor rotation that contributed to the deterioration of the figs, leading to a more rapid release of sugars and other compounds. In this experiment, the ethanol concentration was 9.19 g/L, with a higher lactic acid concentration compared to acetic acid. This is in accordance with fermentations performed on media with sufficient quantities of growth- and metabolism-stimulating compounds and with the results obtained in similar experimental setups by other researchers [8]. At the end of fermentation, some fermentable sugars remained unutilized. In an investigation where the HRTB rotation speed was set to 5 and 7 rpm (Table 2), the highest product concentrations were detected at around 50 h of fermentation (significantly faster compared to the experiments conducted in jars). Additionally, almost all the carbon sources were utilized. The increase in HRTB rotation speed improved mass transfer and media homogeneity, enhancing the efficiency of the bioprocess. However, a higher rotation speed also facilitated better oxygen dissolution, allowing acetic acid bacteria to produce more acetic acid. Consequently, the final acetic acid concentration in experiments with constant HRTB rotation was around 4.0 g/L (Table 2). It was also observed that the increase in HRTB rotation speed led to the disintegration of kefir grains and dried fruits, resulting in a mash-like consistency in the media that differed from its initial state. Based on these results, the lowest rotation speed (3 rpm) was chosen for experiments using the HRTB with interval rotation mode, with the expectation that it would minimize the disintegration of kefir grains. Results obtained using interval HRTB rotation mode (3 min of rotation, followed by 57 min of standing still) are given in Figure 4. As can be seen in Figure 4, significant product formation was observed once all sucrose was hydrolysed. In all three experiments with interval rotation mode, it took around 30 h to hydrolyse all sucrose into glucose and fructose, and these carbon sources stimulated microbial activity. In the first two experiments (3 and 6 min rotation intervals), almost all carbon sources were utilized after 50 h of fermentation. Fructose took slightly longer to be consumed, as it is less preferred by the micro-organisms present in the water kefir grains. Also, some portion of the fermentable sugars came from the dried figs. Namely, 100 g of dried figs contains around 48 g of sugars, out of which 25 g is glucose, and the rest is fructose (Data source: USDA FoodData Central (FDC); fdc.nal.usda.gov, https://fdc.nal.usda.gov/fdc-app.html#/food-details/746768/nutrients, accessed on 20 August 2024). As can be seen in Figure 4B, glycerol concentration significantly increased after fermentable sugars were almost completely consumed (Figure 4A). This phenomenon can be a consequence of the yeasts’ response to the osmotic stress conditions when they are cultivated in media with non-fermentable carbon sources, like ethanol, glycerol, or acetate. In this situation, yeasts will transport glycerol from the cells by a proton symport mechanism into the cultivation media [25].

The highest concentrations of nearly all products were observed when the longest interval rotation time (9 min) was used, reaching peak levels after 80 h. The longer interval rotation time contributed to better broth homogenization and increased mass transfer efficiency, which is evident in the higher acetic acid concentration. By increasing the interval rotation time of the HRTB, the concentration of acetic acid increases when compared to all three experiments with interval rotation mode. However, the increased oxygen concentration likely had a negative effect on some of the micro-organisms present. Namely, most lactic acid bacteria strains are known to be adapted to moderate or lower oxygen concentrations and are generally considered to have an anaerobic metabolism [26,27]. In experiments using constant rotation mode in the HRTB, the increase in the rotation speed generally negatively affected the lactic acid production and lactic acid bacteria growth. This resulted in delayed peak product concentrations and reduced overall productivity. In cases where higher lactic acid concentrations were achieved, lower mannitol concentrations were detected, consistent with the literature indicating that homolactic bacteria are typically poorer producers of mannitol compared to heterolactic bacteria. Water kefir production in the HRTB was significantly faster—at least five times shorter—compared to the static production in jars due to better medium homogeneity in the HRTB resulting from improved mixing conditions. A comparison between water kefir production in the HRTB and a stirred tank bioreactor [13] shows similar bioprocess performance in terms of bioprocess time. Furthermore, in the stirred tank bioreactor, a slightly higher stirrer speed (20 rpm; the lowest measurable value) was used compared to the HRTB (3–7 rpm). This higher stirrer speed likely introduced greater shear stress on the kefir grains, potentially impacting bioprocess performance and efficiency. However, the HRTB has the possibility to work in interval rotation mode which can reduce shear stress on the kefir grains as well as decrease energy consumption on a large scale. During water kefir production in the HRTB, ethanol concentrations were slightly above 1% *v*/*v*, which does not comply with EU regulations [28]. However, this technological challenge in water kefir production using the HRTB can be avoided by optimizing the medium composition, bioreactor operational conditions, and mode together with the optimal composition of the microbial consortium. Based on the obtained results, it is clear that the HRTB is an adequate system for water kefir production, although finer optimization of the HRTB operational conditions is required in order to improve the water kefir quality. Also, it is obvious that kefir grains are not an adequate choice for the standardization of water kefir production. Therefore, future research will focus on defining an optimal microbial consortium (e.g., yeasts, lactic acid bacteria, and acetic acid bacteria) capable of consistently producing water kefir with desired quality. During water kefir production, carbon dioxide and other gasses are also produced. The main gas is carbon dioxide, which is partially dissolved in the water kefir and partially released into the atmosphere. Ensuring the purification and separation of released carbon dioxide and other gases will help in making water kefir production more sustainable by preventing greenhouse gas emissions [28,29]. After standard water kefir production, the grains are isolated from the water kefir by sieving, washed with water, and dried at room temperature. When both kefir grains and dried fruits are used in water kefir production, they can also be separated from the water kefir through sieving [28,29]. However, the separation of these two constituents can be technologically and economically demanding. Therefore, it is expected that in industrial water kefir production, kefir grains and dried fruits remain potential by-products unless additional processing steps are implemented. There are several options for managing the kefir grains and dried fruit residues after separation from the water kefir. One option is to recycle these residues by homogenizing and grinding them for use as sources of growth- and metabolism-stimulating compounds for new water kefir grains. Another option is to separate and purify high-value products (such as vitamins, proteins, peptides, amino acids and polysaccharides) from the residues. Both residues are biologically degradable and, after pretreatment, could potentially be used as medium constituents for biofuel production. Utilizing all by-products from water kefir production in other bioprocesses contributes to making water kefir production an environmentally sustainable process [28,29]. However, it is important to note that implementing a biorefinery concept on an industrial scale is essential to achieving a “zero waste” bioprocess system.

## 5. Conclusions

During this research, the impact of different dried fruits on water kefir production in jars was studied. In all cases, except for the addition of dried figs, relatively slow fermentation dynamics were observed due to the slow hydrolysis of sucrose into glucose and fructose. Notable acetic acid production was usually observed at the end of fermentation, attributed to the presence of ethanol, oxygen, and other nutrients in the cultivation media. Interestingly, similar observations were recorded even when dried figs were added, meaning that acetic acid bacteria and other micro-organisms have adequate quantities of growth- and metabolism-stimulating compounds. In the second phase of this research, the possibility of using an HRTB for water kefir production was explored. By manipulating the HRTB operational conditions (i.e., bioreactor rotation speed and rotation mode), it was possible to produce water kefir with a higher variety of fermentation products that are characteristic of this beverage. The obtained results also show that it is possible to use this bioreactor type for water kefir production. In the case of constant HRTB rotation mode, mass transfer and media homogeneity were improved when HRTB rotation speed was increased. At the same time, acetic acid bacteria activity was enhanced, and consequently, the acetic acid concentration was increased. However, the activity of lactic acid bacteria was reduced, and therefore, the lactic acid concentration was relatively low. The findings also highlight the significant impact of the microbial consortium on fermentation dynamics and duration. The interval rotation mode of the HRTB proved to be a better choice for achieving diverse fermentation product concentration profiles and reducing the overall duration of the bioprocess, indicating greater lactic acid bacteria activity. In conclusion, this study demonstrates that the HRTB can be effectively used for water kefir production. Both constant and interval rotation modes are viable, but while more intense mixing results in better mass transfer, it also significantly affects microbial activity and final product concentrations. The HRTB showed great potential for water kefir production compared to traditional jar production, with a significantly shorter production time (at least five times faster) observed in the HRTB.

## Figures and Tables

**Table 1:** Final pH media value together with all substrates and product concentrations in jar experiments with different types of dried fruits added at the beginning of bioprocess.

Dried Fruit	γ_SUC_ (g/L)	γ_GLU_ (g/L)	γ_FRU_ (g/L)	γ_EtOH_ (g/L)	γ_GLY_ (g/L)	γ_ACET_ (g/L)	γ_LAC_ (g/L)	γ_MANN_ (g/L)	pH
Plums	10.0	0.0	3.0	1.0	0.5	7.0	0.0	4.0	2.9
Raisins	9.0	10.0	30.0	0.1	0.2	0.2	0.0	0.0	2.1
Dates	10.0	10.0	40.0	1.0	0.1	12.0	0.0	0.0	2.0
Papaya	20.0	21.0	50.0	1.3	0.5	6.0	0.0	0.0	2.4
Cranberries	2.0	30.0	52.0	1.0	0.3	6.5	0.0	1.5	2.2
Apricots	0.0	10.0	25.0	0.1	3.0	8.0	0.0	10.0	3.0
10 g of dried figs	0.0	0.0	0.0	0.2	1.0	45.0	6.0	5.0	2.7
20 g of dried figs	0.0	0.0	0.0	0.1	0.2	55.0	12.0	0.0	2.8
30 g of dried figs	0.0	0.0	0.0	12.2	3.0	5.0	3.6	0.4	3.5

**Table 2:** Final pH media value together with all substrates and products concentrations during water kefir production in the HRTB.

Constant Rotation Mode	γ_SUC_ (g/L)	γ_GLU_ (g/L)	γ_FRU_ (g/L)	γ_EtOH_ (g/L)	γ_GLY_ (g/L)	γ_ACET_ (g/L)	γ_LAC_ (g/L)	γ_MANN_ (g/L)	pH
3 rpm	2.20	5.5	2.10	9.19	3.65	3.83	2.17	0.56	3.45
5 rpm	0.77	0.26	0.04	11.72	3.66	3.87	2.16	0.48	3.7
7 rpm	1.04	0.22	0.23	11.30	1.93	4.16	0.29	0.48	3.3
Interval rotation mode									
3, 57 min at 3 rpm	0.39	0.26	0.38	11.52	3.72	0.63	10,14	0.43	3.85
6, 54 min at 3 rpm	0.11	1.17	0.31	11.08	5.39	8.46	8.34	0.38	3.7
9, 51 min at 3 rpm	0.56	0.84	0.30	10.23	4.35	5.90	3.25	0.42	3.7

## Data Availability

The original contributions presented in the study are included in the article. Further inquiries can be directed to the corresponding author.
